# A new ultrasonic process for a renewal of aortic valve decalcification

**DOI:** 10.1186/1476-7120-4-2

**Published:** 2006-01-05

**Authors:** Stéphane Aubert, Eric Voiglio, Lara Chalabreysse, Fadi Farhat, Olivier Jegaden

**Affiliations:** 1 Department of Cardiovascular Surgery, Louis Pradel Cardiologic Hospital, University Hospitals of Lyon, F-69500 Bron, France; 2 Departement of Emergency Surgery, INRETS-UCBL UMR 9002, University Hospitals of Lyon, F-69495 Pierre-Bénite, France; 3 Departement of Pathology, Louis Pradel Cardiologic Hospital, University Hospitals of Lyon, F-69500 Bron, France

## Abstract

**Background:**

Aortic valve decalcification by ultrasound was given up. We evaluated a new ultrasound microhandpiece (Dissectron Penstyle^®^) to rehabilitate this alternative treatment.

**Methods:**

We used under magnifying lenses the ultrasound microhandpiece to decalcify 30 explanted aortic valves. In the cases with embedded calcifications the thin top of the probe could be introduced into the thickness of the leaflet preserving covering layers.

**Results:**

The leaflets were totally decalcified and flexible, and surrounding structures were preserved as assessed by histological examination.

**Conclusion:**

This new approach of ultrasonic aortic valve decalcification gives good in vitro results which allow to consider a clinical evaluation of this procedure.

## Background

The aortic valve debridement by ultrasound in degenerative-calcific aortic stenosis appeared to be an alternative treatment for severe calcified aortic stenosis [1], but was given up because of the high incidence of restenosis and aortic regurgitation [2,3]. The aim of this study was to rehabilitate aortic valve decalcification by a new probe: the microhandpiece Dissectron Penstyle^®^. This probe is more handy and more precise than the previous ones. Therefore the reintroduction of valve decalcification by ultrasound is envisageable.

## Methods

**Figure 1:**
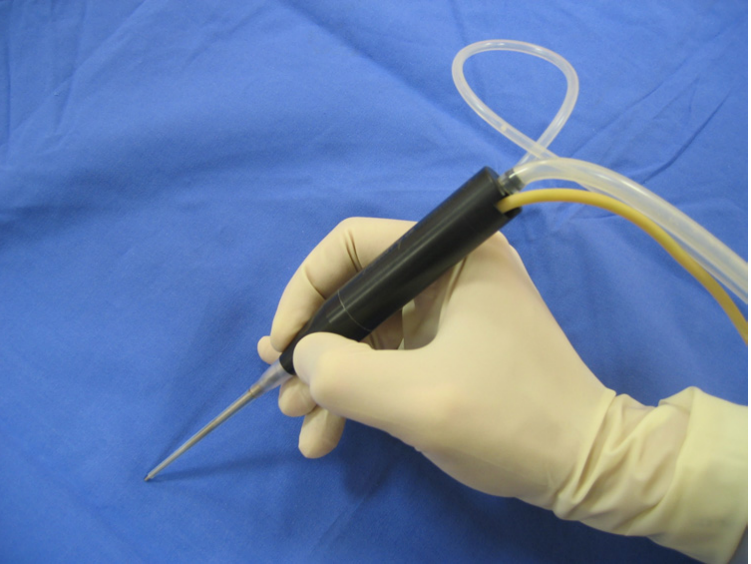
Penstyle micro handpiece with his micro sonotrode: length 192 mm, weight 60 gr, end diameter of the top 1.45 mm, used as a pen.

We used ultrasonic energy to decalcify 30 aortic human tricuspid valves after they had been surgically removed during an aortic valve replacement. The valves had not been selected and were severely calcified in accordance with the necessity of the valve replacement. Ultrasonic decalcification was performed with the Dissectron^® ^Penstyle (Integra NeuroSsciences, Sophia Antipolis, France, EU) used in neuro and general surgery but never used in cardiac surgery. This ultrasonic system consists of a control console with a handheld aspirator that contains a magnetostrictive transducer which converts electrical energy into mechanical motion (piezoelectric technology). The handpiece (Figure 1) has a hollow titanium tip (microsonotrode) that vibrates longitudinally at 30 kHz (comparing with 30 to 60 Hz used in diagnostic echocardiography), thereby fragmenting tissue in contact with its tip proportionally to the water content of the tissue. The end diameter of the Penstyle^® ^was 1.45 mm. The excursion amplitude of the tip could be adjusted from 77 to 154 μm and was set arbitrarily at 110 μm. A side port provided continuous saline solution irrigation and the suspended particles were aspirated through the hollow tip. The irrigation was adjusted to 3 cm^3^/min and the suction on top of the Dissectron^® ^was adjusted to 10–15 mmHg. The irrigating fluid avoided the overheating of the probe. The effects on tissue are the result of the combination of three parameters: ultrasonic energy, irrigation and suction. The ultrasound probe was placed in direct contact with the calcium and the generator was activated by depressing a foot pedal. We used magnifying lenses (3,5 X) to perform the process and the operating surgeon determined the adequacy of decalcification by inspection and palpation of the leaflets. A preliminary study on 10 other valves had allowed us to familiarise with the device and to adjust the settings. The completeness of the leaflet decalcification was studied by palpation and by histopathological examination with the help of a nonparametric grading system (from 1+ to 4+).

## Results

**Figure 2:**
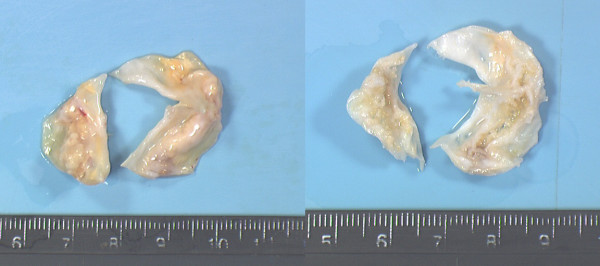
Aortic valve before and after decalcification.

**Figure 3:**
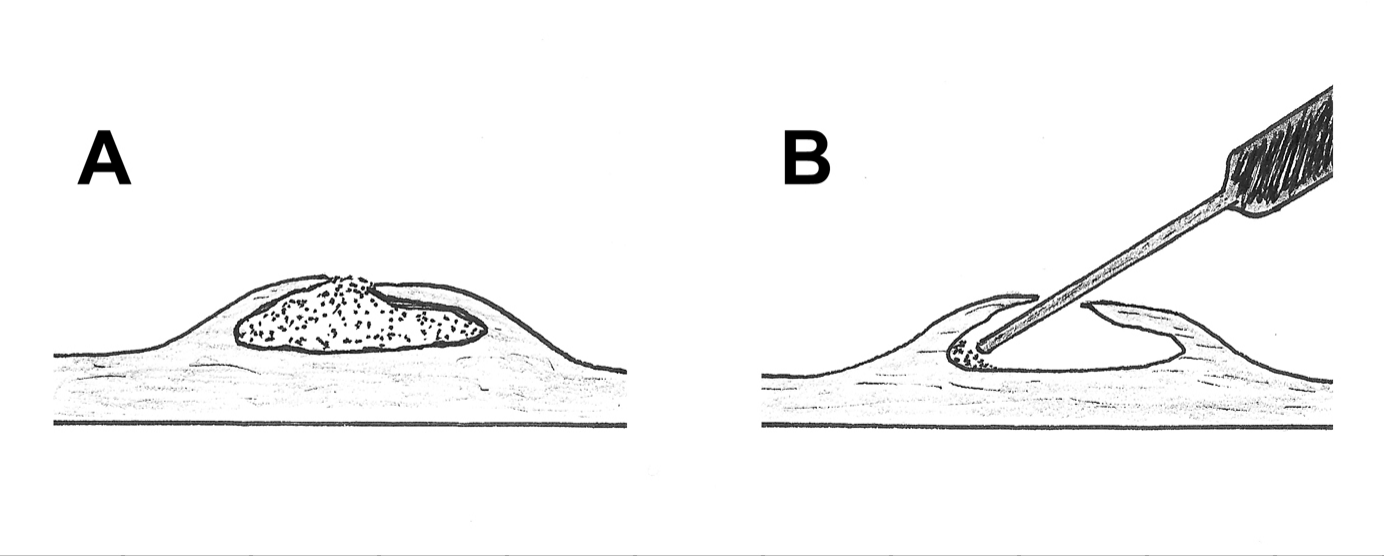
A: Aortic valve before decalcification, an arrow indicates an impressive bulky calcification deposit; B: Aortic valve after decalcification, an arrow indicates a cavity instead of the calcification. This valve is not totally decalcified (3+).

**Figure 4:**
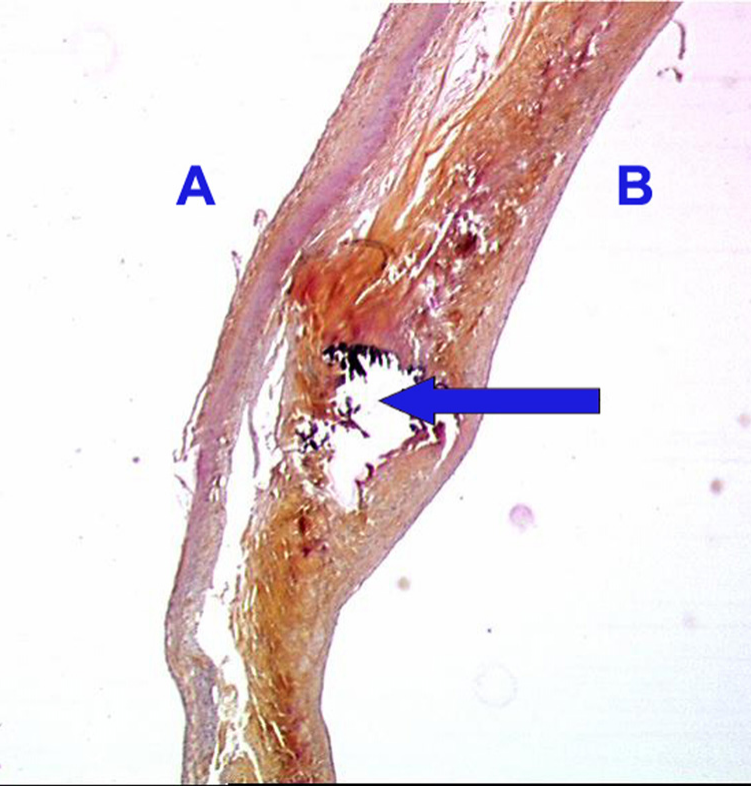
A: Leaflet with embedded calcification, B: The probe into the thickness of the leaflet preserving covering layers.

**Figure 5:**
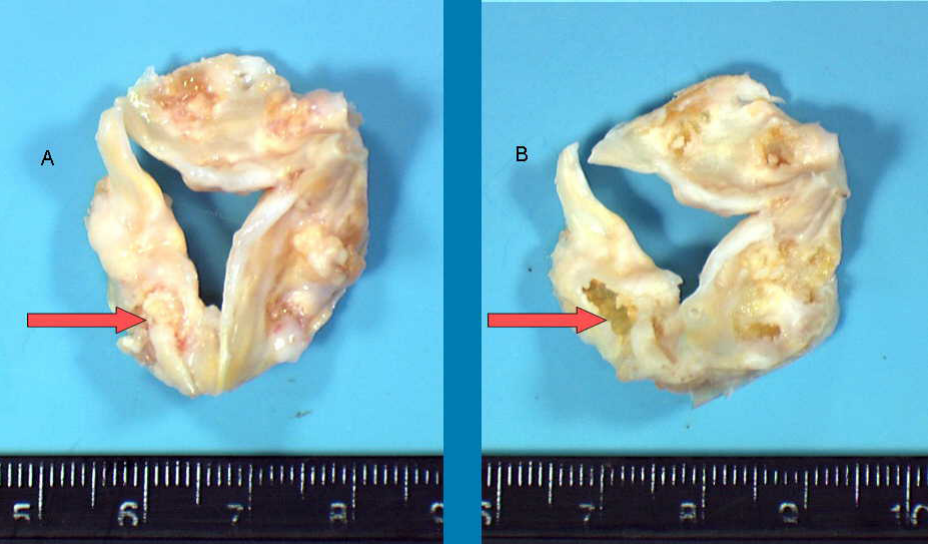
Histological section of an aortic leaflet after decalcification, an arrow indicates a remaining cavity. A: Ventricular side, B: Aortic side.

The Dissectron^® ^handpiece was very ergonomic. The calcium was disintegrated in about 15 minutes in all the 30 cases. At the end of the process all the leaflets were observed as flexible again by palpation assessment. In 26 cases leaflets were totally decalcified (4+) (Figure 2) and in 4 cases almost totally decalcified (3+) (Figure 3). At an amplitude setting of 0.5 (about 110 μm), calcified deposits on the aortic cusps could be easily fragmented and removed, thereby restoring leaflet mobility. It was easier to decalcify aortic valve with calcific lumps only on the aortic surface of the cusps (n = 6). The preliminary study showed us that the optimal angle between the top of the Dissectron^® ^and the valve surface is about 45°. With an angle of 45° we did not provide any perforation in the 30 valves. In the cases with embedded calcification (n = 24) we performed the decalcification by introducing the probe through the aortic surface and directing it inside the thickness of the valve in contact with the calcification (Figure. 4). On microscopic examination, sections of aortic leaflets presented a cavity instead of the calcification and preserved surrounding structures (Figure. 5).

## Discussion

The penstyle handpiece was totally adapted to decalcify aortic valves quickly, with comfort and precision. Although the initial results of the ultrasound decalcification with the previous probes in aortic stenosis were impressive [4,5] two principal problems were observed: early occurrence of aortic insufficiency and significant incidence of restenosis [2,3]. Aortic insufficiency was caused by leaflet retraction and the loss of central coaptation thought to be secondary to the healing response [3]. The aortic restenosis was caused by the accumulation of calcium in the remaining fibrillar structure [6]. The excursion of our tip was 110 μm comparatively to the 154 μm excursion of the Cavitron^® ^used by McBride [7]. As the Penstyle^® ^handpiece decalcifies in better conditions than those previously described [8], with a lower level of mechanical energy, we expected a decreased healing response and consequently a decreased occurrence of aortic insufficiency. There is not any biological effects of our ultrasounds on the normal tissue, therefore we can use our probe safely in a therapeutic approach. The 1.54 mm end diameter of the Penstyle^® ^was extremely precise to decalcify, rendering subsequent damaging of the normal valvular tissue around the calcium limited. The thin top of the probe allowed decalcification within the thickness of the leaflets without destroying the covering layers. The use of magnifying lenses facilitated an accurate procedure. This new approach of decalcification damages the valve as little as possible, so we hope to decrease the risk of early recalcification of the remaining valve. The ultimate test is the longevity of the improved flexibility and function in the patient, so we plan a clinical trial in patients to verify these two parameters. Lastly, we must distinguish two groups: calcific lumps on the aortic surface of the cusps favourable for ultrasound decalcification and embedded calcifications requiring an indispensable complementary treatment of the remaining cavities predisposing to early recalcification. The proposed indication for this new approach is a coronary bypass grafting associated with a stenosis of the aortic valve, when we hesitate to replace the valve. The expected advantage is to avoid a further operation to replace the aortic valve, with all the risks of a redo-operation.

## Conclusion

This new approach of ultrasonic aortic valve decalcification was efficient to decalcify all the valves whatever the degree of calcification. Calcific lumps on the aortic surface of the cusps are favourable for this treatment. The mainly pitfall of our technique is to avoid to decalcify embedded calcifications. Even if it is possible to decalcify this sort of calcification, the remaining cavity will be recalcified very quickly. Only a clinical evaluation of this procedure will demonstrate a decreased risk of both recalcification and secondary aortic regurgitation.

## Competing interests

The author(s) declare that they have no competing interests.

## Authors' contributions

SA did all the research and wrote the paper.

EV had been involved in the research.

LC had been involved in the anatomopathology examination.

FF had been involved in the analysis of the data.

OJ had been involved in the final approval of the version to be published.
